# Simulations of Complex and Microscopic Models of Cardiac Electrophysiology Powered by Multi-GPU Platforms

**DOI:** 10.1155/2012/824569

**Published:** 2012-11-25

**Authors:** Bruno Gouvêa de Barros, Rafael Sachetto Oliveira, Wagner Meira, Marcelo Lobosco, Rodrigo Weber dos Santos

**Affiliations:** ^1^Computational Modeling, Federal University of Juiz de Fora, 36036-900 Juiz de Fora, MG, Brazil; ^2^Computer Science, Federal University of São João del-Rei, 36307-352 São João del-Rei, MG, Brazil; ^3^Computer Science, Federal University of Minas Gerais, 31270-901 Belo Horizonte, MG, Brazil

## Abstract

Key aspects of cardiac electrophysiology, such as slow conduction, conduction block, and saltatory effects have been the research topic of many studies since they are strongly related to cardiac arrhythmia, reentry, fibrillation, or defibrillation. However, to reproduce these phenomena the numerical models need to use subcellular discretization for the solution of the PDEs and nonuniform, heterogeneous tissue electric conductivity. Due to the high computational costs of simulations that reproduce the fine microstructure of cardiac tissue, previous studies have considered tissue experiments of small or moderate sizes and used simple cardiac cell models. In this paper, we develop a cardiac electrophysiology model that captures the microstructure of cardiac tissue by using a very fine spatial discretization (8 **μ**m) and uses a very modern and complex cell model based on Markov chains for the characterization of ion channel's structure and dynamics. To cope with the computational challenges, the model was parallelized using a hybrid approach: cluster computing and GPGPUs (general-purpose computing on graphics processing units). Our parallel implementation of this model using a multi-GPU platform was able to reduce the execution times of the simulations from more than 6 days (on a single processor) to 21 minutes (on a small 8-node cluster equipped with 16 GPUs, i.e., 2 GPUs per node).

## 1. Introduction

 Heart diseases are responsible for one third of all deaths worldwide [1]. Cardiac electrophysiology is the trigger to the mechanical deformation of the heart. Therefore, the knowledge of cardiac electrophysiology is essential to understand many aspects of cardiac physiological and physiopathological behavior [2]. Computer models of cardiac electrophysiology [3, 4] have become valuable tools for the study and comprehension of such complex phenomena, as they allow different information acquired from different physical scales and experiments to be combined to generate a better picture of the whole system functionality. Not surprisingly, the high complexity of the biophysical processes translates into complex mathematical and computational models. Modern cardiac models are described by nonlinear system of partial differential equations (PDEs) that may result in a problem with millions of unknowns. 

Mathematical models for cell electrophysiology are a key component of cardiac modeling. They serve both as standalone research tools, to investigate the behavior of single cardiac myocytes, and as an essential component of tissue and organ simulation based on the so-called bidomain or monodomain models [4]. The cell models can be written as a general non-linear system of ordinary differential equations (ODEs) and may vary in complexity from simple phenomenological models [5] (based on two variables) to complex models describing a large number of detailed physiological processes [6] (based on 40 to 80 differential variables). Simple models focus on the genesis of action potential (AP), that propagates from cell to cell and generates an electric wave that propagates on the heart. Complex models account not only for the genesis of AP but also describe how this phenomenon is related to cardiac homeostasis and to different sub-cellular components, such as cell membrane's ion channels. Advances in genetics, molecular biology, and electrophysiology experiments have provided new data and information related to the structure and function of ion channels. The Markov Chain (MC) model formalism has been increasingly used to describe both function and structure of ion channels. MC-based models have enabled simulations of structural abnormalities due to genetic diseases and drug-biding effects on ion channels [7–9]. Unfortunately, these modern cardiac myocyte models pose different challenges to both numerical methods, due to the stiffness of the ODEs introduced by MCs, and to high performance computing, due to the size of the problems, since the number of differential variables rises from a couple to near a hundred [10].

On the tissue level, the bidomain model [4] is considered to be the most complete description of the electrical activity. This nonlinear system of PDEs can be simplified to the so-called monodomain model, which may be less accurate but less computationally demanding than the bidomain model. Unfortunately, large scale simulations, such as those resulting from the discretization of an entire heart, remain a computational challenge. In addition, key aspects of cardiac electrophysiology, such as slow conduction, conduction block, and saltatory or sawtooth effects, demand sub-cellular discretization for the solution of the PDEs and nonuniform, heterogeneous tissue electric conductivity. These aspects of cardiac electrophysiology are strongly related to cardiac arrhythmia, reentry, fibrillation or defibrillation, and have been the research topic of many studies [11–20].

However, the demand of sub-cellular discretization for the solution of the PDEs and nonuniform, heterogeneous tissue electric conductivity have prevented the study of the aforementioned phenomena on large-scale tissue simulations. In addition, due to the high computational costs associated with the simulations of these microscopic models of cardiac tissue, previous works have adopted simple myocyte models, instead of modern MC-based models [6, 10].

In this work, we present a solution for this problem based on multi-GPU platforms (clusters equipped with graphics processing units) that allows fast simulations of microscopic tissue models combined with modern and complex myocyte models. The solution is based on merging two different high-performance techniques. We have previously investigated for cardiac modeling: cluster computing based on message passing communications (MPI) [21–24] and GPGPU (General-purpose computing on graphics processing units) [25–30]. We developed a two-dimensional model that is based on the previous work of Spach and collaborators [11, 17] that accounts for the microstructure of cardiac tissue, gap junction heterogeneous distribution, and discretizations of 8 *μ*m. This microscopic tissue model was combined with the model of Bondarenko et al. [6] which is a modern and complex myocyte model based on MCs. Our parallel implementation of this model using a multi-GPU platform was able to reduce the execution times of the simulations from more than 6 days (on a single processor) to 21 minutes (on a small 8-node cluster equipped with 16 GPUs, that is, 2 GPUs per node). As a result, using this very fast parallel implementation we were able to simulate the formation of spiral waves, a form of self-sustained reentrant activity strongly associated with cardiac arrhythmia. To the best of our knowledge, this is the first time spiral waves are simulated using a cardiac model that accounts for both the microstructure of cardiac tissue and a modern and complex myocyte model.

## 2. Methods

### 2.1. Modeling Cardiac Microstructure

**Figure 1:**
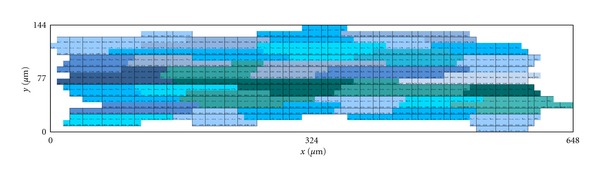
Basic unit of cardiac myocyte distribution based on a total of 32 cells. Cells are displayed in different alternating colors along the *x*-axis. The basic unit spans a total of 648 *μ*m in the longitudinal direction versus 144 *μ*m in the transversal direction.

We developed a two-dimensional model that is based on the previous work of Spach and collaborators [11, 17] that accounts for the microstructure of cardiac tissue, gap junction heterogeneous distribution, and discretizations of 8 *μ*m × 8 *μ*m. A basic template for myocyte connections was developed and is presented in Figure 1. This basic unit accounts for the connection of a total of 32 cardiac myocytes with different shapes and numbers of neighboring cells. The mean and SD (standard deviation) values for cell length and width are 120.9 ± 27.8 *μ*m and 18.3 ± 3.5 *μ*m, respectively. These values are close to those reported in the literature: [31] (length = 140 *μ*m and width = 19 *μ*m), [32] (length = 134 *μ*m and width = 18 *μ*m), and [20] (length = 100 *μ*m and width = 17.32 *μ*m). On average, each cell connects to other 6 neighboring myocytes. Our two-dimensional model considers a homogeneous depth *d* = 10 *μ*m [11, 17].

**Figure 2:**
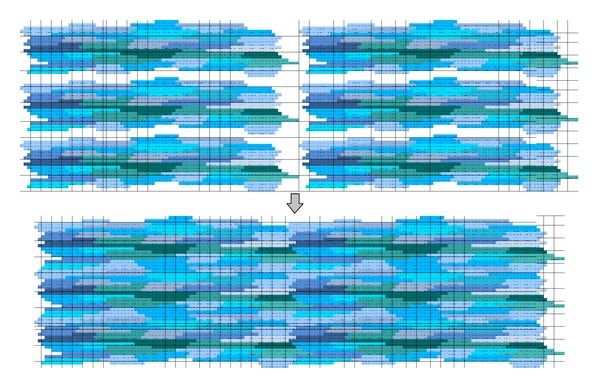
Six basic units being combined to form a larger tissue.

This basic unit was created in such a way that it allows the generation of larger tissue preparations via the connections of multiple instances of it.  Figure 2 presents how this can be achieved.

**Figure 3:**
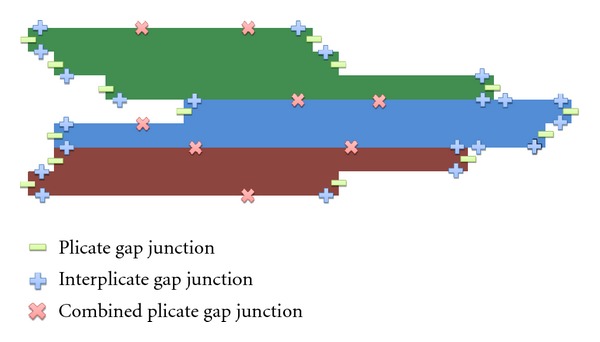
In this work, there are only 5 possible types of connections between neighboring volumes that are membrane, which indicated no-flux between neighboring volumes; cytoplasm, which indicates that the neighboring volumes are within the same cell; three possible types of gap junctions, plicate, interplicate, and combined plicate. For the simulations presented in this work, the gap junction distribution of the basic template unit was manually chosen to reproduce the distribution presented before in [11, 17]. This figure presents an example of how different gap junctions are distributed in three neighboring myocytes that belong to the basic unit.

Figure 3 presents an example of how the connections between different myocytes can be arranged. The code was developed in a flexible way, so that it allows the user to set up for each discretized volume Vol_*i*,*j*_ (with  area = *h* × *h*) conductivity or conductance values for the north (*σ*_*x*_*i*,*j*+1/2__), south (*σ*_*x*_*i*,*j*−1/2__), west (*σ*_*x*_*i*−1/2,*j*__), and east (*σ*_*x*_*i*+1/2,*j*__) volume faces. These can be any nonnegative values. In this work, we set the discretization *h* to 8 *μ*m. In addition, based on the work of Spach and collaborators [11, 17], we chose only 5 possible types of connections between neighboring volumes that are membrane (*σ*_*m*_ = 0.0), cytoplasm (*σ*_*c*_ = 0.4 *μ*S/*μ*m), gap junction plicate (*G*_*p*_ = 0.5 *μ*S), interplicate (*G*_*i*_ = 0.33 *μ*S), and combined plicate (*G*_*c*_ = 0.062 *μ*S), where we use *σ* for conductivity and *G* for conductance. For the simulations presented in this work, the distribution of the different gap junctions within the 32 myocytes was not randomly generated. Instead, the gap junction distribution of the basic template unit was manually chosen to reproduce the distribution presented before in [11, 17]. With this setup and conductivity values we found that conduction velocity along the fibers was around 410 *μ*m/ms (LP) and was 130 *μ*m/ms transversal to fiber direction (TP). This results in a ratio LP/TP of 0.32, which is close to the conduction ratio reported in [11]. 

### 2.2. The Heterogeneous Monodomain Model

Action potentials propagate through the cardiac tissue because the intracellular space of cardiac cells is electrically coupled by gap junctions. In this work, we do not consider the effects of the extracellular matrix. Therefore, the phenomenon can be described mathematically by a reaction-diffusion type partial differential equation (PDE) called monodomain model, given by

(1) βCm∂V(x,y,t)∂t+βIion(V(x,y,t),η(x,y,t))  =∇·(σ(x,y)∇V(x,y,t))+Istim(x,y,t),∂η(x,y,t)∂t=f(V(x,y,t),η(x,y,t)),

where *V* is the variable of interest and represents the transmembrane potential, that is, the difference between intracellular to extracellular potential; **η** is a vector of state variables that also influences the generation and propagation of the electric wave and usually includes the intracellular concentration of different ions (K^+^, Na^+^, Ca^2+^) and the permeability of different membrane ion channels; *β* is the surface-volume ratio of heart cells; *C*_*m*_ is the membrane capacitance, *I*_ion_ the total ionic current, which is a function of *V* and a vector of state variables **η**; *I*_stim_ is the current due to an external stimulus, **σ** is the monodomain conductivity tensor. We assume that the boundary of the tissue is isolated, that is, no-flux boundary conditions (**n** · *σ*∇*V* = 0 on ∂*Ω*).

In this work, the modern and complex Bondarenko et al. model [6] that describes the electrical activity of left ventricular cells of mice was considered to simulate the kinetics of *I*_ion_ in (1). The Bondarenko et al. model (BDK) was the first model presented for mouse ventricular myocytes [6]. The ionic current term *I*_ion_ in this model consists of the sum of 15 transmembrane currents. In short, Bondarenko's model is based on a ordinary differential equation (ODE) with 41 differential variables that control ionic currents and cellular homeostasis. In this model, most of the ionic channels are represented by Markov chains (MCs).

### 2.3. Numerical Discretization in Space and Time

The finite volume method (FVM) is a mathematical method used to obtain a discrete version of partial differential equations. This method is suitable for numerical simulations of various types of conservation laws (elliptical, parabolic, or hyperbolic) [33]. Like the finite element method (FEM), the FVM can be used in several types of geometry, using structured or unstructured meshes, and generates robust numerical schemes. The development of the method is intrinsically linked to the concept of flow between regions or adjacent volumes, that is, it is based on the numerical calculation of net fluxes into or out of a control volume. For some isotropic problems discretized with regular spatial meshes, the discretization obtained with the FVM is very similar to the one obtained with the standard finite difference method (FDM).

This section presents a brief description of the FVM application to the time and spatial discretization of the heterogeneous monodomain equations. Detailed information about the FVM applied to the solution of monodomain can be found in [34, 35].

#### 2.3.1. Time Discretization

The reaction and diffusion parts of the monodomain equations were split by employing the Godunov operator splitting [36]. Therefore, each time step involves the solution of two different problems: a nonlinear system of ODEs

(2) ∂V∂t=1Cm[−Iion(V,η)+Istim],∂η∂t=f(V,η),

and a parabolic PDE

(3) β(Cm∂V∂t)=∇·(σ∇V).

Since the spatial discretization of our model, *h*, is extremely small, the CFL [37] condition that assures numerical stability is very restrictive. Therefore, for the PDE we used the unconditionally stable implicit Euler scheme. The time derivative presented in (3), which operates on *V* is approximated by a first-order implicit Euler scheme as follows:

(4) ∂V∂t=Vn+1−VnΔtp,

where *V* ^*n*^ represents the transmembrane potential at time *t*_*n*_ and Δ*t*_*p*_ is the time step used to advance in time the partial differential equation.

For the discretization of the nonlinear system of ODEs, we note that its stiffness demands very small time steps. For simple models based on Hodgkin-Huxley formulation, this problem is normally overcome by using the Rush-Larsen (RL) method [38]. However, for the most modern and complex models that are highly based on MCs, the RL method seems to be ineffective in terms of allowing larger time steps during the numerical integration. For the case of the Bondarenko et al. model, we tested both methods, Euler and RL, and both demanded the same time step, Δ*t*_*o*_ = 0.0001 ms for stability issues. Since the RL method is more expensive per time step than the Euler method, in this work, we used the simple explicit Euler method for the discretization of the nonlinear ODEs.

However, as already indicated above, we use different time steps for the solution of the two different uncoupled problems, the PDE and the ODEs. Since we use an unconditionally stable method for the PDE, the time step Δ*t*_*p*_ could be much larger than that used for the solution of the nonlinear system of ODEs, Δ*t*_*o*_ = 0.0001 ms. In this work, we use Δ*t*_*p*_ = 0.01 ms, that is, a hundred times larger than Δ*t*_*o*_. This has not introduced any significant numerical error. We calculated the L2 relative error for the transmembrane potential between a solution that uses the same time step for both the ODE and the PDE, Δ*t*_*o*_ = Δ*t*_*p*_ = 0.0001 ms, *V*_*m*_ref__ and a solution that uses Δ*t*_*o*_ = 0.0001 ms and Δ*t*_*p*_ = 0.01 ms, *V* as follows:

(5) error=∑i=1nt∑j=1nv(V(i,j)−Vmref(i,j))2∑i=1nt∑j=1nvVmref(i,j)2,

where *nt* is the number of time steps and *nv* is the total number of discretized volumes. For the simulation of a tissue of size 0.5 × 0.5 cm during 20 ms (stimulus at the center of the tissue), the error found was 0.01%.

#### 2.3.2. Spatial Discretization

The diffusion term in (3) must be discretized in space. For this we will consider the following:

(6) J=−σ∇V,

where **J** (*μ*A/cm^2^) expresses the density of intracellular current flow and

(7) ∇·J=−Iv.

In this equation, *I*_*v*_ (*μ*A/cm^3^) is a volumetric current and corresponds to the left-hand side of (3), serving as the base for this finite volume solution.

For the space discretization, we will consider a two-dimensional uniform mesh, consisting of regular quadrilaterals (called “volumes”). Located in the center of each volume is a node. The quantity of interest *V* is associated with each node of the mesh.

After defining the mesh geometry and dividing the domain in control volumes, the specific equations of the FVM can be presented. Equation (7) can be integrated spatially over an individual volume *V*_*i*,*j*_ of size *h* ^2^ *d*, leading to

(8) ∫Ω∇·J dv=−∫ΩIv dv.

Applying the divergence theorem yields

(9) ∫Ω∇·J dv=∫∂ΩJ·ξ→ ds,

where $\vec{\xi }$ is the unitary normal vector to the boundary ∂*Ω*. Then, we have

(10) ∫∂ΩJ·ξ→ ds=−∫ΩIv dv.

Finally, assuming that *I*_*v*_ represents an average value in each particular quadrilateral, and substituting (3) in (10), we have

(11) β(Cm∂V∂t)|(i,j)=−∫∂ΩJi,j·ξ→ dsh2d.

For this particular two-dimensional problem, consisting of a uniform grid of quadrilaterals with side *h*, the calculation of **J**_*i*,*j*_ can be subdivided as a sum of flows on the following faces:

(12) ∫∂ΩJi,j·ξ→ ds=(Ixi+1/2,j−Ixi−1/2,j+Iyi,j+1/2−Iyi,j−1/2),

where *I*_*x*_*m*,*n*__ and *I*_*y*_*m*,*n*__ are calculated at faces ((*m*, *n*) = (*i* + 1/2, *j*), (*i* − 1/2, *j*), (*i*, *j* + 1/2), or (*i*, *j* − 1/2)) as follows. For the case in which we have defined a conductivity value at face (*m*, *n*), for instance the intracellular, or cytoplasm conductivity, *σ*_*c*_, as described in Section 2.1, we have

(13) Ixm,n=−σc(m,n)∂V∂x|(m,n)hd,Iym,n=−σc(m,n)∂V∂y|(m,n)hd.

For the case in which we have defined a conductance value at face (*m*, *n*), for instance a gap junction conduction *G*, as describes in Section 2.1, we have:

(14) Ixm,n=−G(m,n)ΔxV|(m,n),Iym,n=−G(m,n)ΔyV|(m,n).

Using centered finite difference, we have for (13)

(15) ∂V∂x|(i+1/2,j)=Vi+1,j−Vi,jh,∂V∂y|(i,j+1/2)=Vi,j+1−Vi,jh.

For (14), we have

(16) ΔxV|(i+1/2,j)=Vi+1,j−Vi,j,ΔyV|(i,j+1/2)=Vi,j+1−Vi,j.

Equations for ∂*V*/∂*x*∣_(*i*−1/2,*j*)_, ∂*V*/∂*y*∣_(*i*,*j*−1/2)_, Δ_*x*_ *V*∣_(*i*−1/2,*j*)_ and Δ_*y*_ *V*∣_(*i*,*j*+1/2)_ can be obtained analogously.

Rearranging and substituting the discretizations of (4) and (12) in (11) and decomposing the operators as described by (2), and (3) yields

(17) (σi+1/2,j+σi−1/2,j+σi,j+1/2+σi,j−1/2+α)Vi,j∗−σi,j−1/2Vi,j−1∗ −σi+1/2,jVi+1,j∗−σi,j+1/2Vi,j+1∗−σi−1/2,jVi−1,j∗=αVi,jn,

(18) CmVi,jn+1−Vi,j∗Δto=−Iion(Vi,j∗,ηn),

where *α* = (*βC*_*m*_ *h* ^2^)/Δ*t*_*p*_, *n* is the current step, ∗ is an intermediate step, and *n* + 1 is the next time step. In addition *σ* can stand for any of the gap junction conductance (*G*_*p*_, *G*_*i*_, *G*_*c*_) divided by the depth *d* or for any conductivity value (*σ*_*c*_, *σ*_*m*_) defined for each volume face as described in Section 2.1. This defines the equations for each finite volume Vol_*i*,*j*_. First we solve the linear system associated with (17) to advance time by Δ*t*_*p*_ and then we solve the nonlinear system of ODEs associated with (18) *N*_*o*_ times until we have *N*_*o*_Δ*t*_*o*_ = Δ*t*_*p*_.

### 2.4. Parallel Numerical Implementations

Large scale simulations, such as those resulting from fine spatial discretization of a tissue, are computationally expensive. For example, when an 8 *μ*m discretization is used in a 1 cm × 1 cm tissue and the Bondarenko et al. model (BDK), which has 41 differential variables, is used as cardiac cell model, a total of 1250 × 1250 × 41 = 64,062,500 unknowns must be computed at each time step. In addition, to simulate 100 ms of cardiac electrical activity, 64 millions of unknowns of the nonlinear systems of ODEs must be computed one million times (with Δ*t*_*o*_ = 0.0001 ms) and the PDE with 1.5 million of unknowns must be computed ten thousand times.

To deal with this high computational cost, two distinct tools for parallel computing were used together: MPI and GPGPU.

#### 2.4.1. Cluster Implementation

The *cluster* implementation is a parallel implementation tailored to cluster of CPUs. The *cluster* implementation uses the PETSc [39] and MPI [40] libraries. It uses a parallel conjugate gradient preconditioned with ILU(0) (with block Jacobi in parallel) to solve the linear system associated with the discretization of the PDE of the monodomain model. More details about this parallel implementation can be found in our previous works on this topic [21–24].

To solve the non-linear systems of ODEs, the explicit Euler method was used. This is an embarrassingly parallel problem. No dependency exists between the solutions of the different systems of ODEs of each finite volume Vol_*i*,*j*_. Therefore, it is quite simple to implement a parallel version of the code: each MPI process is responsible for computing a fraction *Np* of the total number of volumes of the simulation, where *Np* is the number of processes involved in the computation.

#### 2.4.2. Multi-GPU Implementation

In our *multi-GPU* implementation, we have decided to keep the cluster approach for the solution of the linear system associated with the discretization of the PDE of the monodomain model. Therefore, *multi-GPU* also solves the discretized PDE with the parallel conjugate gradient preconditioned with ILU(0) (with block Jacobi in parallel) available in the PETSc library.

However, we have accelerated the solution of the systems of ODEs by using multiple GPUs. This is a different strategy from those we have used before when the full Bidomain equations (elliptic PDE, parabolic PDE, and systems of ODEs) were completely implemented in a single GPU [30], or the full Monodomain equations (parabolic PDE and system of ODEs) were completely implemented in a single GPU [25, 26, 29].

The motivation for choosing a different strategy is based on several reasons. As presented in [29], the monodomain model can be accelerated using a single GPU by 35-fold when compared to a parallel OpenMP [41] implementation running on a quad-core computer. However, this final speedup obtained by the GPU comes from a near 10-fold speedup for the solution of the PDE and a near 450-fold speedup for the solution of the nonlinear systems of ODEs. Nowadays, as manycore architecture evolves, one may easily find in the market a single computer equipped with 64 processing cores. Therefore, we believe that solving the PDE on these new machines with traditional MPI or OpenMP-based parallel implementations may outperform a single GPU implementation. On the other side, for the parallel solution of the nonlinear systems of ODEs a single GPU still easily outperforms these new manycore-based computers. This bring, us to focus GPU implementations to the parallel solutions of the millions of nonlinear systems of ODEs. A second motivation is related to the preconditioners that can be easily and efficiently implemented for the conjugate gradient method in GPUs. For the bidomain equations, efficient geometric multigrid preconditioners [30] were implemented in a single GPU, and sophisticated algebraic multigrid preconditioners [42] were implemented in a multi-GPU platform. However, both implementations are only viable for the solution of the linear system associated with the elliptic PDE of the bidomain equations. Multigrid preconditioners are too expensive and turns out to be an inefficient option for the solution of the parabolic PDE, which is the PDE type of the monodomain model. Until now, the cheap but inefficient *w*-Jacobi preconditioner has been the best choice for GPU implementations when it concerns the solution of the parabolic PDE [29, 42]. However, it is well known that incomplete LU (ILU) preconditioners combined with block Jacobi or additive Schwarz domain decomposition methods [23] greatly outperform Jacobi-like preconditioners on cluster computing for the solution of the PDE of the monodomain model. This argument favors cluster-like implementations as the best choice for the parallel solution of the parabolic PDE of the monodomain model (see [43] and the references cited therein). Finally, a third and last motivation is related to the particular problem we propose to investigate in this work: models that reveal the microstructure of cardiac tissue. Another recent work presented an implementation for the bidomain model for multi-GPU platforms [44]. Both PDEs and systems of ODEs were implemented on GPUs using explicit methods, Jacobi relaxation, and explicit Euler, respectively. We note that for our particular microscopic tissue model with spatial discretization of 8 *μ*m, the approach of using an explicit and cheap solver for the PDE would be very inefficient due to the severe stability restrictions imposed by the CFL conditions [37]. Therefore, once more, this argument also favors cluster- like implementations based on implicit methods for the parallel solution of the parabolic PDE of the monodomain model.

Our *multi-GPU* implementation uses CUDA [45] to implement the numerical solution of the BDK cardiac cell model. The CUDA model extends the C programming language with a set of abstractions to express parallelism, that is, CUDA includes C software development tools and libraries to hide the GPGPU hardware details from programmers that can focus on important issues of the parallelism of their code rather than dealing with unfamiliar and complicated concepts from computer graphics in order to explore the computational power of GPUs for general purpose computation.

In order to run an application, the programmer must create a parallel function called kernel. A kernel is a special C function callable from the CPU but executed on the GPU simultaneously by many threads. Each thread is run by a GPU stream processor. They are grouped into blocks of threads or just blocks. The blocks can be one-, two-, or three-dimensional. A set of blocks of threads form a grid, that can be one- or two-dimensional. When the CPU calls the kernel, it must specify how many blocks and threads will be created at the GPU to execute the kernel. The syntax that specifies the number of threads that will be created to execute a kernel is formally known as the execution configuration and is flexible to support CUDA's hierarchy of threads, blocks of threads, and grids of blocks. Since all threads in a grid execute the same code, a unique set of identification numbers is used to distinguish threads and to define the appropriate portion of the data they must process. These threads are organized into a two-level hierarchy composed by blocks and grids and two unique coordinates, called *blockId* and *threadId*, are assigned to them by the CUDA runtime system. These two built-in variables can be accessed within the kernel functions and they return the appropriate values that identify a block and thread, respectively. All the threads within a single block are allowed to synchronize with each other via a special barrier operator, called *syncthread*, and have access to a high-speed, per-block shared memory which allows interthread communication. Threads from different blocks in the same grid can coordinate their execution only through the use of atomic global memory operations. No assumptions are made about the execution order of thread blocks, which means that a kernel must execute correctly no matter the order in which blocks are scheduled by the hardware to run.

Some additional steps must be followed to use the GPU: (a) the device must be initialized; (b) memory must be allocated in the GPU and data transferred to it; (c) the kernel is then called. After the kernel have finished its execution, results are transferred back to the CPU.

Two kernels have been developed to solve each of the systems of ODEs related to BDK model. The first kernel is responsible for setting the initial conditions of the systems of ODEs, whereas the second one integrates the systems of ODEs at each time step.

Both kernel implementations were optimized in many different ways. The state variables of *M* cardiac cells were stored in an array called SV, whose size is equal to *MN*_eq_, where *N*_eq_ is the number of differential equations of the ionic model (in this work, *N*_eq_ is equal to 41). The SV array was organized in such a way that the first *M* entries correspond to the first state variable, followed by *M* entries of the next state variable, and so on. Moreover, for all ionic models, the first *M* entries of the SV array correspond to the transmembrane potential *V*. During the solution of the systems of PDEs, after the integration of the ODEs systems, the transmembrane potential of each node should be passed to the PETSC solver. Due to the memory organization chosen for the SV array, this is a straightforward task since, as stated before, the *M* first entries of the array correspond to the transmembrane potential *V* of each node. This organization allows us to avoid extra memory transactions between CPU and GPU, improving performance. Another implementation choice that impact performance positively was the way the SV array has been allocated. The SV array was allocated in global GPU memory using the *cudaMallocPitch* routine from the CUDA API. This routine may pad the allocation in order to ensure that corresponding memory addresses of any given row will continue to meet the alignment requirements for the coalescing operations performed by the hardware. In short, a strict coalescing requires that thread *j* out of *n* threads has to access data *u*[*j*] if *u*[0] is accessed by thread 0, that is, each thread should perform data access by stride *n*. Therefore, in the first kernel, to set the initial conditions, each thread sets the values of all its state variables. The kernel that solves the system of ODEs operates similarly, that is, each thread computes and updates its state variables writing to the right position in memory that corresponds to their variables. In addition, the second kernel was optimized to use as much as local memory operations as possible.

**Figure 4:**
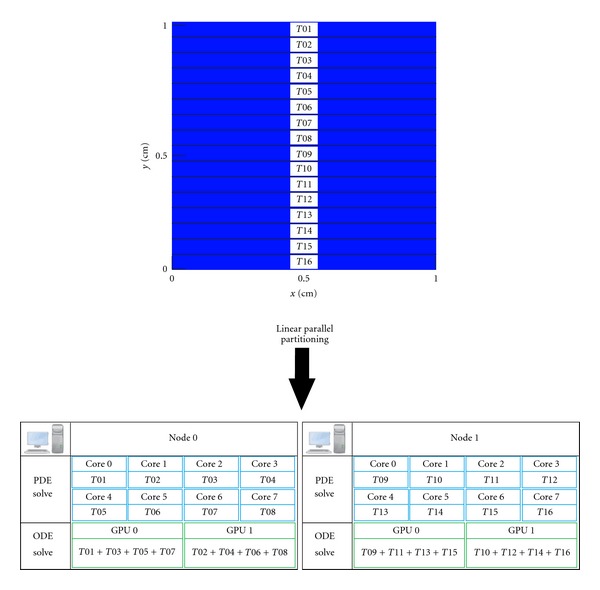
Linear parallel decomposition of tissue. Example for the case of two nodes (each with 8 cores and 2 GPU devices, that is, a total of 16 CPU cores and 4 GPU devices). Each CPU core processes one task. Four tasks are assigned to each GPU device. For instance, at node 0, GPU 0 processes tasks *T*_1_, *T*_3_, *T*_5_, and *T*_7_, and GPU 1 the tasks *T*_2_, *T*_4_, *T*_6_, and *T*_8_.

Pure domain decomposition was used for parallelism. The tissue domain was linearly decomposed on *Np* nonoverlapping subdomains (or *Np* tasks, *T*_1_ to *T*_*Np*_, see Figure 4), where *Np* is the number of MPI processes or processing cores. The parallel solution of the PDE is implemented via PETSc (see [21]), with each processing core *p* responsible for updating the variables associated to subdomain *T*_*p*_. In our computational environment each machine or node has more CPU cores (8) than GPUs (2). Therefore, for the solution of the ODEs each GPU device will be responsible for processing more than one task. The tasks assigned with one node are distributed to the GPUs in a round-robin fashion. For example, if *Np* = 16 and we have two machines (each with 8 cores and 2 GPU devices), Figure 4 presents how the tissue domain will be partitioned. Four tasks would be assigned to each GPU device. For instance, at node 0, GPU 0 would process tasks *T*_1_, *T*_3_, *T*_5_, and *T*_7_, GPU 1 the tasks *T*_2_, *T*_4_, *T*_6_, and *T*_8_.

For the solution of the ODEs, both sequential and parallel (CUDA) codes used single precision. For the solution of the PDE we have used double precision. For the case of monodomain simulations, we have shown in [25] that the use of single precision in CUDA does not affect the numerical precision of the solver.

## 3. In Silico Experiments, Computational ****Environment, and Metrics

The simulations were performed using the microscopic model with spatial discretization of 8 *μ*m and heterogeneous conductivity values as described in Section 2.1. The values used for *β* and *C*_*m*_ were set to 0.14 cm^−1^ and 1.0 *μ*F/cm^2^, respectively. The time step used to solve the linear system associated with (17) was set to Δ*t*_*p*_ = 0.01 ms and to solve the nonlinear system of ODEs associated to (18) was set with Δ*t*_*o*_ = 0.0001 ms.

Three different tissue setups were used to test our model and parallel implementations: a cardiac tissue of 0.5 cm × 0.5 cm size that was stimulated in the center and was executed for 10 ms, a cardiac tissue of 1.0 cm × 1.0 cm size that was stimulated in the center and was executed for 10 ms, and a cardiac tissue of 1.0 cm × 1.0 cm that was stimulated using the S1-S2 protocol to generate a spiral wave, a form of self-sustained reentrant activity strongly associated with cardiac arrhythmia.

Our experiments were performed on a cluster of 8 SMP computers. Each computer contains two Intel E5620 Xeon quad-core processors and 12 GB of RAM. All nodes run Linux version 2.6.18 – 194.17.4.el5. The codes were compiled with gcc 4.1.2 and CUDA 3.2. Each node contains two Tesla C1060. The Tesla C1060 card has 240 CUDA cores and 4 GB of global memory.

All tests were performed three times. The average execution time, in seconds, is then used to calculate the speedup, defined as the sequential execution time divided by the parallel execution time.

## 4. Results

**Figure 5:**
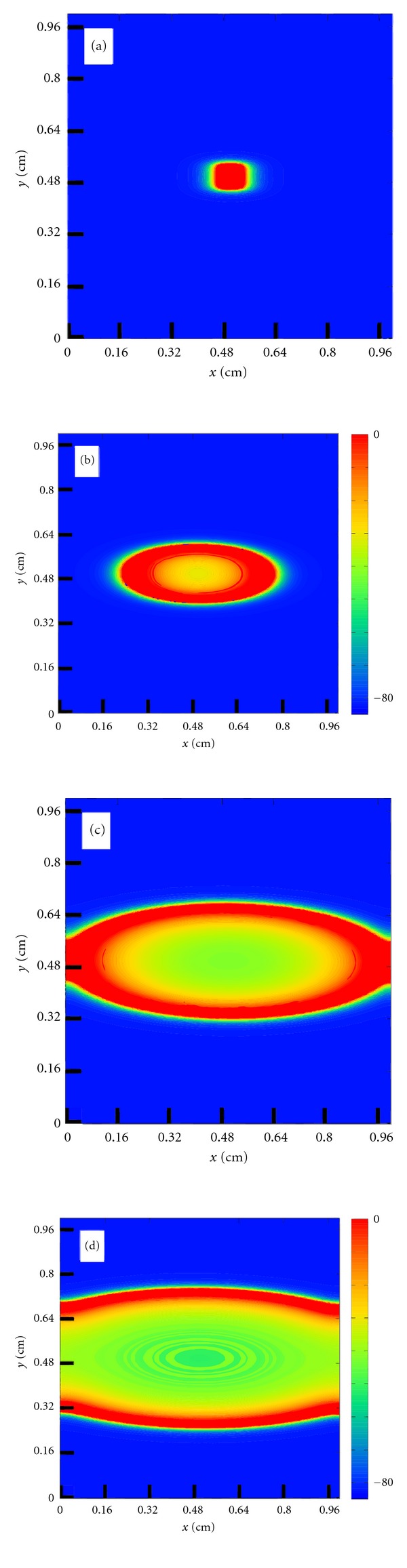
Action potential propagation (transmembrane potential) after a central stimulus on a tissue of size 1 cm × 1 cm for different time instants. (a) *t* = 1 ms, (b) *t* = 7 ms, (c) *t* = 13 ms, and (d) *t* = 20 ms.

**Figure 6:**
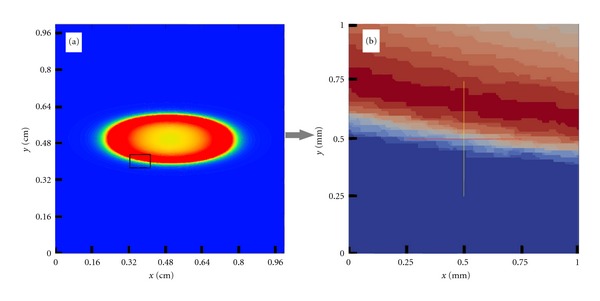
(a) Transmembrane potential at *t* = 7 ms after a central stimulus on a tissue of size 1 cm × 1 cm. (b) Microscopic details revealing the discrete nature of AP propagation, that is, the influence of cardiac microstructure on a large tissue size simulation.

Figure 5 presents the propagation of a central stimulus on the tissue of size 1 cm × 1 cm for different time instants. As expected, macroscopically, the propagation looks very smooth and continuous. However, when highlighting a smaller region of size 1 mm × 1 mm, see Figure 6, we can already observe the discrete nature of propagation, that is, the influence of the cardiac microstructure on the propagation of action potentials.

Table 1 presents the results obtained by the parallel implementations for the experiment with a square tissue of 0.5 cm × 0.5 cm. As one can observe, the time spent solving the ODEs is responsible for near 90% of the execution time. It can also be observed that although the obtained speedups with the *cluster* are respectable and almost linear (near 61 with 64 cores), the total execution time remains high. With respect to the *multi-GPU *implementation, the results are much better. It must be stressed that although 64 cores where used in this simulation, only 16 GPGPU devices where available for executing the simulation, so 8 processes share 2 GPGPU devices per machine. As one can observe, the obtained speedup was huge, about 343 times faster than a single core processor. The execution time drops from 1.6 days (using one processing core) to only 6.7 minutes (using the 8-node multi-GPU platform). 

Table 2 presents the results obtained by the parallel implementations for the experiment with a square tissue of 1.0 cm × 1.0 cm. Once again, the speedups obtained with the *cluster* implementation, were almost linear (61 with 64 cores). With respect to the *multi-GPU* implementation the results are much better. The speedup was huge, about 420 times faster than a single core processor. The execution time drops from more than 6 days (using one processing core) to only 21 minutes (using the 8-node multi-GPU platform). We can also observe that the *multi-GPU* implementation was near 7 times faster than the *cluster* implementation when running on the 8 computers.

**Figure 7:**
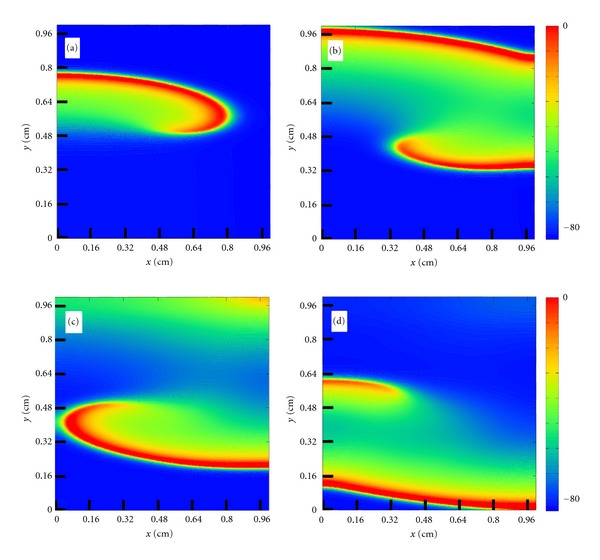
Spiral wave formation after an S1-S2 stimulus protocol at different time instants: (a) *t* = 80 ms, (b) *t* = 100 ms, (c) *t* = 112 ms, and (d) *t* = 120 ms.

As a result, using this very fast parallel implementation, we were able to simulate the formation of spiral waves, a form of self-sustained reentrant activity strongly associated with cardiac arrhythmia, see Figure 7. To the best of our knowledge, this is the first time spiral waves are simulated using a cardiac model that accounts for both the microstructure of cardiac tissue and a modern and complex myocyte model. After a couple of tries using the S1-S2 protocol to find the correct vulnerable window, we managed to generate a sustained spiral wave using this cardiac model that accounts for both the microstructure of cardiac tissue and a modern and complex myocyte model. The whole process took less than one day (around 13 hours with each simulation taking between 3 and 7 hours). Without our multi-GPU parallel implementation, this process would have taken 227 days using a single core computer or near 4 days using our cluster implementation running with 64 cores but without the GPUs.

## 5. Discussion and Future Works

Our results show that our *multi-GPU* parallel implementation described in Section 2.4.2 was able to significantly accelerate the numerical solution of a cardiac electrophysiology model that captures the microstructure of cardiac tissue (using a very fine spatial discretization) and is based on a very modern and complex cell model (with Markov chain formulation that has been extensively used for the characterization of ion channels). Speedups around 420 times were obtained, reducing execution times from more than 6 days (using one processing core) to only 21 minutes (using the 8-node multi-GPU platform). The hybrid *Multi-GPU *parallel implementation presented in this work is even more attractive if one considers that the architectures of GPUs and multicore processors continue to evolve on a fast pace.

Nevertheless, we believe our parallel implementation can be further improved. For instance, in the current implementation, the CPU cores are idle while waiting for the results of the nonlinear ODEs that are being computed by the GPU devices. For future work, we intend to evaluate different load balancing techniques to better distribute the parallel tasks between GPU devices and CPU cores and make a more efficient use of all the computational resources. Another possible improvement is related to the multilevel parallelism introduced for the solution of the bidomain equations [24] that combines task parallelism (via pipeline) and data parallelism (via data decomposition). We believe a similar combination of data and task parallelism could be also exploited for the solution of the monodomain equations to further enhance the parallel efficiency of our algorithms.

Recent studies that focus on the discrete or discontinuous nature of AP propagation have avoided the computational challenges that arise from microscopic models via the development and use of discrete models, where each cardiac myocyte is represented by a single point connected with the neighboring myocytes by different conductivities [46, 47]. This description has allowed the study of the effects of randomly distributed conductivities in the conduction velocity and on the formation of reentry patterns on cardiac tissue. Discrete models were introduced by Keener in [48] to describe the electrical propagation in a 1*D* cable of *nc* connected cells for the case of low gap-junctional coupling. In this model, the cells are assumed to be isopotential. Therefore, only gap junction conductances are considered for the connection of neighboring myocytes, that is, cytoplasmic resistance is considered to be insignificant. Recently, we have compared discrete and microscopic models for a 1*D* cable of connected cells [49]. We have shown that the numerical results obtained by the discrete model are similar to those obtained by the heterogeneous microscopic model for the case of low gap-junctional coupling (1%–10% of normal coupling). However, the discrete model failed for the case of normal gap-junctional coupling or moderate reduced gap-junctional coupling (50%–100% of normal coupling). The two-dimensional microscopic model developed in this work will allow us to further compare these two approaches (detailed microscopic models versus discrete models) and to better understand the benefits and limitations of each one of them. In addition, we hope that our microscopic model may also suggest ways to better develop discrete models, which are computationally less expensive than the detailed microscopic ones.

## 6. Conclusion

In this paper, we developed a cardiac electrophysiology model that captures the microstructure of cardiac tissue by using a very fine spatial discretization and uses a very modern and complex cell model based on Markov chains for the characterization of ion channel's structure and dynamics. To cope with the computational challenges, the model was parallelized using a hybrid approach: cluster computing and GPGPUs. Different *in silico* tissue preparations were used in this work for the performance tests. We have shown that in all cases, our parallel multi-GPU implementation was able to significantly reduce the execution times of the simulations, for instance, from more than 6 days (on a single processor) to 21 minutes (on a small 8-node cluster equipped with 16 GPUs, that is, 2 GPUs per node). We believe that this new parallel implementation paves the way for the investigation of many open questions associated with the complex and discrete propagation nature of action potentials on cardiac tissue.

## Figures and Tables

**Table 1:** Average execution time and speedup of parallel implementations for a tissue of 0.5 cm × 0.5 cm. The execution times are presented in seconds.

Parallel implementation	Cores	Total Time	ODE time	PDE time	Speedup
Cluster	1	137,964	132,590	5,264	—
Cluster	8	18,492	17,210	1,262	7.5
Cluster	16	9,922	9,316	595	13.9
Cluster	32	4,198	3,884	311	32.9
Cluster	64	2,283	2,087	191	60.4

Multi-GPU	64 + 16 GPUs	401.84	209.4	187	343

**Table 2:** Average execution time and speedup of parallel implementations for a tissue of 1.0 cm × 1.0 cm. The execution times are presented in seconds.

Parallel implementation	Cores	Total Time	ODE time	PDE time	Speedup
Cluster	1	546,507	523,331	23,177	—
Cluster	64	8,934	8,313	607	61.2

Multi-GPU	64 + 16 GPUs	1,302	682	611	420
